# High opsin diversity in a non-visual infaunal brittle star

**DOI:** 10.1186/1471-2164-15-1035

**Published:** 2014-11-28

**Authors:** Jérôme Delroisse, Esther Ullrich-Lüter, Olga Ortega-Martinez, Sam Dupont, Maria-Ina Arnone, Jérôme Mallefet, Patrick Flammang

**Affiliations:** Biology of Marine Organisms and Biomimetics, Research Institute for Biosciences, University of Mons, Avenue du Champs de Mars 6, 7000 Mons, Belgium; Museum für Naturkunde, Invalidenstr. 43, 10115 Berlin, Germany; Department of Biological and Environmental Science, The Sven Lovén Centre for Marine Sciences – Kristineberg, University of Gothenburg, 45178 Fiskebäckskil, Sweden; Stazione Zoologica Anton Dohrn, Cellular and Developmental Biology, Villa Comunale, 80121 Naples, Italy; Laboratory of Marine Biology, Earth and Life Institute, Catholic University of Louvain, Louvain-La-Neuve, Place Croix du Sud 3, bt L7.06.04, 1348 Louvain-la-Neuve, Belgium

**Keywords:** Opsin, Echinodermata, Ophiuroidea, Photoreception, Genome, Transcriptome

## Abstract

**Background:**

In metazoans, opsins are photosensitive proteins involved in both vision and non-visual photoreception. Echinoderms have no well-defined eyes but several opsin genes were found in the purple sea urchin (*Strongylocentrotus purpuratus*) genome. Molecular data are lacking for other echinoderm classes although many species are known to be light sensitive.

**Results:**

In this study focused on the European brittle star *Amphiura filiformis*, we first highlighted a blue-green light sensitivity using a behavioural approach. We then identified 13 new putative opsin genes against eight *bona fide* opsin genes in the genome of *S. purpuratus*. Six opsins were included in the rhabdomeric opsin group (r-opsins). In addition, one putative ciliary opsin (c-opsin), showing high similarity with the c-opsin of *S. purpuratus* (Sp-opsin 1), one Go opsin similar to Sp-opsins 3.1 and 3.2, two basal-branch opsins similar to Sp-opsins 2 and 5, and two neuropsins similar to Sp-opsin 8, were identified. Finally, two sequences from one putative RGR opsin similar to Sp-opsin 7 were also detected. Adult arm transcriptome analysis pinpointed opsin mRNAs corresponding to one r-opsin, one neuropsin and the homologue of Sp-opsin 2. Opsin phylogeny was determined by maximum likelihood and Bayesian analyses. Using antibodies designed against c- and r-opsins from *S. purpuratus*, we detected putative photoreceptor cells mainly in spines and tube feet of *A. filiformis*, respectively. The r-opsin expression pattern is similar to the one reported in *S. purpuratus* with cells labelled at the tip and at the base of the tube feet. In addition, r-opsin positive cells were also identified in the radial nerve of the arm. C-opsins positive cells, expressed in pedicellariae, spines, tube feet and epidermis in *S. purpuratus* were observed at the level of the spine stroma in the brittle star.

**Conclusion:**

Light perception in *A. filiformis* seems to be mediated by opsins (c- and r-) in, at least, spines, tube feet and in the radial nerve cord. Other non-visual opsin types could participate to the light perception process indicating a complex expression pattern of opsins in this infaunal brittle star.

**Electronic supplementary material:**

The online version of this article (doi:10.1186/1471-2164-15-1035) contains supplementary material, which is available to authorized users.

## Background

Light is one of the most important selective evolutionary forces for living organisms [1]. In metazoans, luminous information is mainly detected through photosensitive proteins, the opsins, which are involved in both vision and non-visual photoreception [2]. In echinoderms, the new genetic information which was made available by the publication of the complete genome of the purple sea urchin *Strongylocentrotus purpuratus*[3] generated an increased interest in echinoid larval and adult photoreception [4–10]. The sea urchin genome contains genes coding for at least six opsins of which four (Sp-opsin 4, Sp-opsin 1, Sp-opsin 3.1, Sp-opsin 3.2, see Additional file 1) are homologous to the rhabdomeric (r), ciliary (c) and Go opsins required for light perception in metazoans [4]. As most species lack true eyes (defined as organs for spatial vision that compare light levels in several directions simultaneously using shadowing, reflection, or refraction [11]), echinoderm photoreception has usually been considered as diffuse, at the level of either the integument or the nervous system [12]. However, in adult *S. purpuratus*, r-opsins were immunodetected in tube feet and c-opsins in spines, pedicellariae, tube feet and some portions of epidermis [4, 5, 9, 10]. Photoreceptors are therefore not as uniformly scattered as researchers postulated at first but are clustered in specific organs, which together, would constitute a complex “photosensory machinery” [4, 5, 9, 10]. In addition, some sea urchin larvae could rely on an opsin-mediated light detection system to perform vertical migrations (Hp-opsin1 also called encephalopsin, see Additional file 1[7]). The diversity of opsins therefore seems to be related to different photoreceptor cell types and to a large variety of light-driven behaviours [9, 13–15].

Information for other echinoderm classes remains limited. Yet, behavioural, morphological and molecular studies showed that at least some species have advanced photoreception capabilities [10, 13, 16–18]. In Holothuroidea and Crinoidea, almost no information is available except for sea cucumbers of the order Apodida in which eye-like structures have been described [19, 20]. Sea stars are the only echinoderm class to possess eye-like structures, the optic cushions at the extremity of each arm [21–25]. Opsins were detected in these organs [9, 25] but also in the aboral integument [26] and in aboral spines [10]. When the optic cushions are removed, light sensitivity is not impaired but directional locomotion is affected [27–29]. Therefore extraocular photoreception also occurs in echinoderms exhibiting eye-like structures. In ophiuroids, many species have been documented to be photosensitive and some species change colour in response to light [12]. Cobb and Moore [30] described specific epidermal ciliated cells as putative photoreceptors, a suggestion later contested by Hendler and Byrne [17]. In some species, it was also suggested that the arm dorsal ossicles might focalise light, in the manner of microlenses, on presumptive internal photoreceptor cells [17, 31]. Johnsen [25] detected “rhodopsin-like” proteins in the arms of the brittle star *Ophioderma brevispinum* by western blot using anti-mammal rhodopsin antibodies, and Ullrich-Lüter *et al.*[10] highlighted the expression of a ciliary opsin-like protein in the spines by immunohistochemistry using an anti-sea urchin c-opsin antibody in two other ophiuroid species, *Amphiura filiformis* and *Ophiocomina nigra*.

This study is focused on the European ophiuroid *A. filiformis* which is a dominant species on most sublittoral soft bottoms in Europe [32]. This species is characterised by an infaunal lifestyle and can reach densities of up to 3000 ind/m^2^[32, 33]. Avoiding visual predation by fishes and crustaceans [34–36], individuals of *A. filiformis* stay almost entirely in the mud during the day and feed on suspended particles at night by extending two arms in the water column [37–40]. Receptors on the arms are thought to detect the optimal conditions for feeding such as currents, food and light [40]. This easily accessible brittle star was recently used as an emerging model species in several molecular studies [41–43]. *A. filiformis* would so constitute a judicious choice for the study of opsin-based photoreception in brittle stars on the one hand, and in infaunal echinoderms on the other hand. Genome and transcriptome analyses made it possible to highlight putative opsin genes and their expression in the arms of *A. filiformis*. Moreover, specific antibodies directed against sea urchin r- and c-opsins were used to localise the homologous opsins in the arms of *A. filiformis*. Here the unexpected high opsin diversity and the complex opsin expression pattern in a burrowing species are reported. What these results suggest about extraocular light perception functions and opsin-based photoreception evolution in brittle stars and echinoderms is then discussed.

## Methods

### Organism sampling

Adult individuals of *A. filiformis* (O.F. Muller, 1776) were collected in the vicinity of the Sven Lovén Centre for Marine Sciences - Kristineberg (Fiskebäckskil, Sweden) in summer at a depth of 30 m. The brittle stars were carefully rinsed out of the sediment, and intact specimens were kept in sediment with running deep seawater (DSW, 14°C, salinity 32, pH_T_ 8.0).

### Behavioural study of light perception in *A. filiformis*

Behavioural experiments were conducted at the Sven Lovén Centre for Marine Sciences in August 2010. During the experiment, day/night cycle was manipulated using specific wavelengths/colours. Dimmed monochromatic LED lamps (1 W) were used for the experiments and their spectra were first evaluated with a minispectrometer (Hamamatsu Photonics K.K. TM – VIS/NIR: C10083CA, Hamamatsu-City, Japan). The experiment was simultaneously conducted in 5 separate aquaria (22×15 cm and 12 cm high) with specific light treatment (white light, green light ≈ 515 nm, blue light ≈ 465 nm, red light ≈ 630 nm and a control with no light) and continuous flow of DSW (around 9 liters/hour). Each aquarium contained 18 brittle stars with intact arms placed on a 5 cm layer of sieved sediment. Light intensity was adjusted with neutral filters to match the natural conditions encountered *in situ*. The light intensity measured above the sediment with a luminometer was 1.5 × 10^12^ photons/s.m^2^ (5.000.000 RLU). Before the experiment, animals were acclimated in the aquaria for 3 days. During this acclimatisation period, a 13 h (day)/11 h (night) photoperiod was used with artificial white light as daylight (day between 7 am and 8 pm). The same photoperiod was conserved during the experiment and the white daylight was replaced by the specific colour treatments. The activity of *A. filiformis* was estimated by counting the number of arms visible in the water during the day (9 am, 1:30 pm) and the night (10 pm, 1:30 am) using photography under infrared light. Recordings were performed for 8 days. For each light treatment (corresponding to one aquarium), mean day and night data were calculated. A parametric unpaired student’s test that compares the means of the two groups (day and night) was used to confront the number of arms protruding from the sediment during day and night time. Beforehand, variance homogeneities were evaluated using the F test. The normal distributions of the values for each group (day and night for each treatment) were also tested using various normality tests (KS normality tests, D’Agostino and Pearson omnibus normality tests, Shapiro-Wilk normality tests). Statistical analyses were performed using GraphPad Prism 5.0 (GraphPad Software, San Diego, CA, USA; http://www.graphpad.com).

### *In silico*analyses

**Figure 1:**
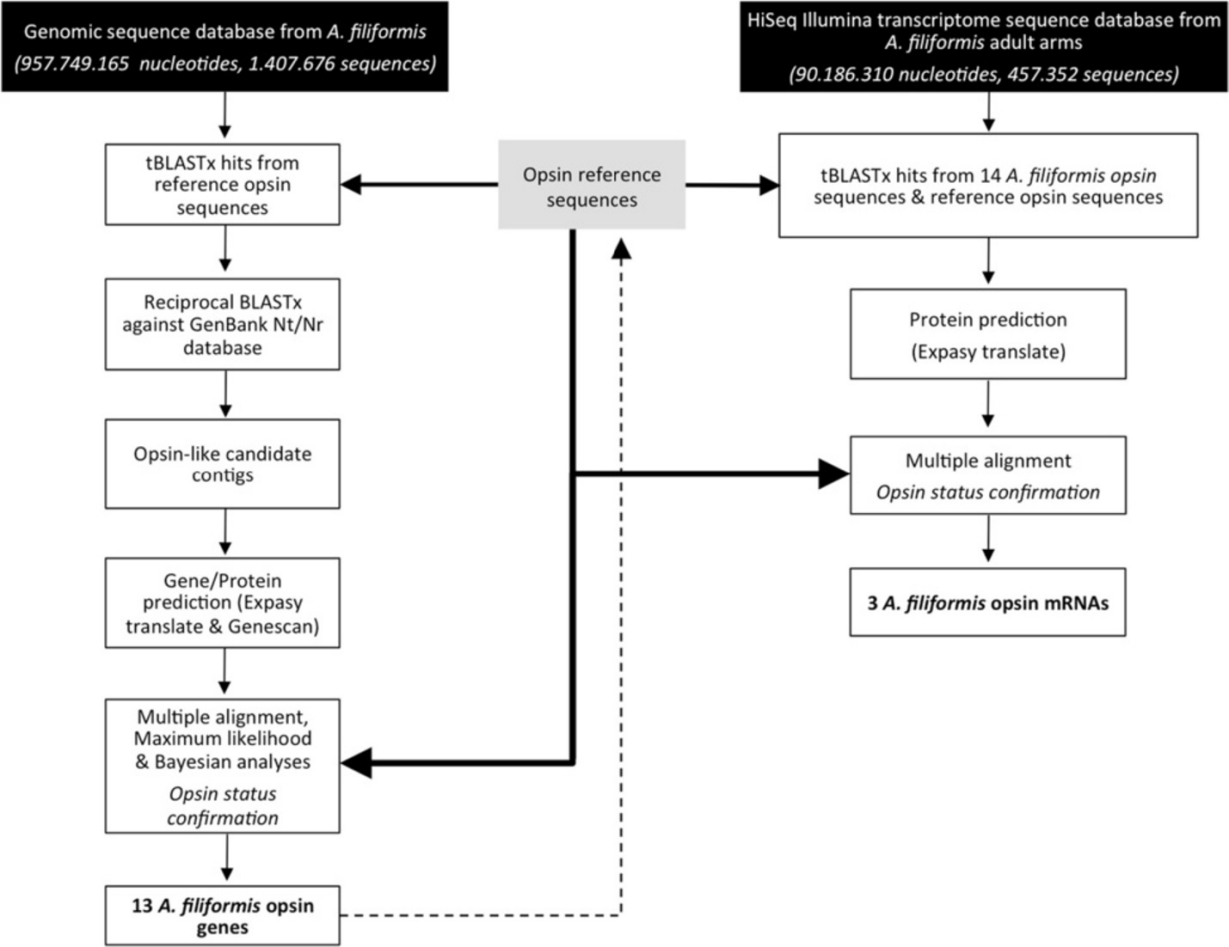
**Outline of the search pipeline used to identify putative opsin sequences in our datasets (see** **Methods** **for details).** For reference sequences, see Additional file 2.

The search pipeline used to identify and characterise putative opsin sequences in *A. filiformis* is presented in Figure 1. Both genomic and transcriptomic data have been used (see below).

### Genome analysis

**Figure 2:**
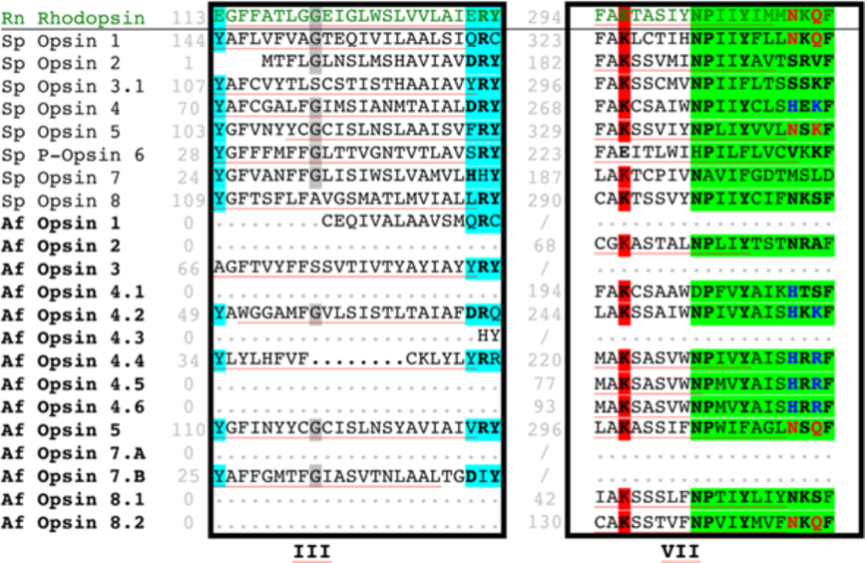
**Amino acid similarity (%) between Af-opsins and Sp-opsins (+ Rn Rhodopsin).** Similarity measurements were conducted between each Af-opsin and all reference opsins (Sp, Rn) on the basis of a local alignment. Trimming was performed on the local alignment. Each similarity estimation is depending on the length of the local alignment. Values framed in red indicate best similarities.

A draft genome of *A. filiformis* (contact person: Olga Ortega-Martinez; http://www.cemeb.science.gu.se/research/target-species-imago+/amphiura-filiformis/) was used to search for opsin genes using a tBLASTx/BLASTx approach. A dataset of opsin sequences (see Additional file 1A) including sea urchin opsins (*S. purpuratus,* Sp-opsins 1, 2, 3.1, 3.2, 4, 5, 6, 7, and 8, [4, 10]), a cephalopod (*Loligo vulgaris*) rhodopsin (typical r-opsin), and a mammalian (*Rattus* norvegicus) rhodopsin (typical c-opsin) was used in local tBLASTx (tFASTx 36.3.4, [44]) searches on the *A. filiformis* genome assembly (957749165 residues and 1407676 sequences). Candidate matches were used as queries in a reciprocal BLASTx (2.2.25, [45]) search against online databases (All non-redundant GenBank CDS translations + PDB + SwissProt + PIR + PRF excluding environmental samples from WGS projects, 7.766.063.076 total letters and 22.586.145 sequences) in order to highlight sequences with high similarity to opsins. *In silico* translation (Expasy, translate tool [46]) and gene structure prediction (GENSCAN Web Server http://genes.mit.edu/GENSCAN.html, [47]) were performed on the opsin-like sequences retrieved from the draft genome of *A. filiformis*. Sequence alignment was used to find *bona fide* opsin sequences after transmembrane helices and Schiff base lysine identification. Secondary structure prediction – in particular transmembrane helix – was done using MENSAT online tool [48]. A multiple amino-acid alignment of putative opsins was performed on total sequences using Seaview 4.2.12 [49] and the muscle algorithm [50]. Aligned residues were highlighted by similarity group conservation (defined by the software) and similarity comparisons were calculated in Mega v5.2.1 [51, 52] (see caption of Figure 2 for more details). Sequence alignments also made it possible to identify opsin characteristic features such as the Schiff base residue, the counterion, the amino acid triad present in the helix involved in the G protein contact, and putative disulfide bond sites. Predicted molecular weights for the opsins were calculated using the “Compute pI/Mw tool” on the ExPASy Proteomics Server [46].

### Transcriptome analysis

A HiSeq 2000 Illumina transcriptome was recently obtained from multiple arms of *A. filiformis* adult individuals collected during day-time in November 2012 (J.D., unpublished observations/data). Arm tissues were separated from the disc to avoid contamination from the digestive tract. Tissue samples were immediately placed in TRIzol^®^ solution for RNA extraction using the RiboPure™ RNA extraction kit (Ambion AM1924). Extractions were performed according to the manufacturer’s protocols. Library preparation and sequencing were performed by BGI (Beijing Genomics Institute, China - http://www.genomics.cn). On the basis of *A. filiformis* and reference (sea urchin and metazoans) opsin sequences, local tBLASTn (2.2.26) (and BLASTn for the brittle star gene sequences) searches were used to target opsin mRNA sequences expressed in the arms tissues [45]. The Illumina derived short read files are available at the NCBI Sequence Read Archive (SRA) under the study accession number SRR1523743.

### Phylogenetic analyses

All new putative opsin sequences of *A. filiformis* were included in phylogenetic analyses based on the previously mentioned alignment. Echinoderm opsin sequences, either published or available in online databases, were added to the analysis. Metazoan opsin sequence data were collected as references from open-access NCBI databases (http://www.ncbi.nlm.nih.gov) and are listed in the supplementary data (Additional file 1). The sequences chosen for the analysis were selected in order to have representative candidates for all opsin classes (ciliary opsins [c-opsins], rhabdomeric opsins [r-opsins], Go-coupled opsins, neuropsins, peropsins and retinal G-protein coupled receptors [RGR opsin]; following [53–55]). Cnidarian and ctenophore opsins were not included in our phylogenetic analysis as they are specific to these lineages [54, 56]. Trees were constructed using truncated alignment/sequences (295 amino acids – mainly the conserved 7TM core of the protein) where the opsin extremities were discarded to avoid unreliably aligned regions. N-terminal sequences upstream of residue 68 and C-terminal sequences downstream of residue 357 of the Opsin 1 of *S. purpuratus*, taken as a reference protein, were therefore excluded. A sequence of a non-opsin GPCR (i.e. melatonin receptor) was chosen as outgroup following [55, 56]. The phylogeny was constructed using the PHYML tool [57, 58] from SeaView 4.2.12 software [49], which allows for the fast estimation of large data sets within a maximum likelihood (ML). A best-fit model analysis was performed using Mega v5.2.1 (following the AIC criteria) [51, 52] and “Wheland and Goldman model of protein evolution” was found to be the best suited and was used for the analyses (WAG, [59]). Branch support values were estimated as bootstrap proportions from 500 PhyML bootstrap replicates. We also performed a Bayesian analysis with MrBayes 3.2 [60] using the GTR+G model. This model was recently reported to be more reliable for opsin phylogeny estimation than the WAG model [55]. Four independent runs of 2,000,000 generations were performed reaching a standard deviation value inferior to 0,01 according to [10, 60]. The resulting phylogenies were compared to the trees generated in previous studies [4, 10, 26, 53–55].

### Whole-mount immunofluorescence

In order to detect putative c- and r-opsins in *A. filiformis*, purified polyclonal antibodies directed against the C-terminal tail of Sp-Opsin 1 (residues 314–361) and C-terminal tail of Sp-Opsin 4 (residues 295–394), respectively, were used. The antibody development is detailed in [9, 10]. The animals were anesthetised using 7% MgCl in a 1:1 mixture of filtered sea water and distilled water and dissected arm tips (the body region most likely to be light-sensitive considering the burrowing way of life of *A. filiformis*) were directly transferred to a 4% solution of paraformaldehyde in filtered sea water or phosphate buffered saline (PBS: 0.05 M PB/0.3 M NaCl, pH 7.4) for 30–60 minutes at room temperature. Fixed samples were decalcified through treatment with 2% ascorbic acid/0.15 M NaCl for 2–6 days on a slow rotator at room temperature. They were then rinsed in PBS and blocked in the same buffer containing 0.25% bovine serum albumin, 0.1% triton X-100 and 0.05% NaN_3_ for 30 minutes at room temperature. Anti-acetylated α- tubulin (SIGMA), anti-Sp-opsin 4 and anti-Sp-opsin 1 were diluted in PBS with final dilutions of 1:250, 1:50 and 1:50, respectively. After an overnight incubation at 4°C, tissues were rinsed in PBS and then incubated in a 1:500 dilution of Alexa Fluor488 conjugated goat anti-rabbit IgG and Alexa Fluor568 conjugated goat anti-mouse IgG (Molecular Probes) for 2 hours at room temperature. After several washes in PBS, specimens were mounted in an antifading medium containing a Glycerin/PBS mixture and examined using a Leica TCS-SPE, a Leica SP5 or a Zeiss 510 Meta confocal microscope. Projections shown in the present study were produced by recording confocal image stacks and projecting them in the z-axis using MacBiophotonics, ImageJ or Fiji. The specificity of the immunofluorescent labelling was confirmed by control experiments using exactly the same procedure without using the primary or secondary antibodies.

## Results

### Spectral photosensitivity estimation in *A. filiformis*

**Figure 3:**
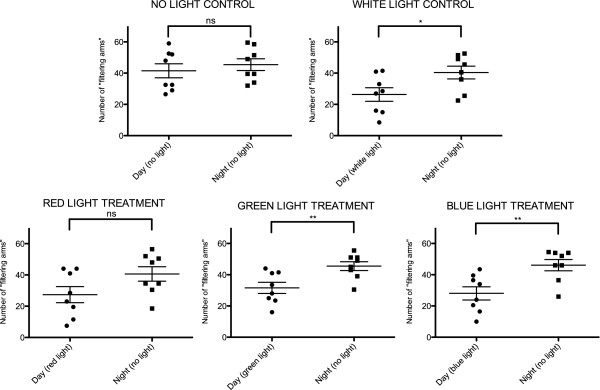
**Scatter-plots comparing the number of arms observed out of the sediment during the day (specific colour treatments) and the night (means ± S.E.M.).** Different treatment conditions (white daylight, no daylight, red/green/blue daylights) are represented in each graph. */**Significant difference between day and night for a particular treatment, ^ns^No significant difference.

Behavioural experiments were performed on adult brittle stars to confirm (i) their light sensitivity and (ii) highlight their general spectral sensitivity. A significant decrease in arm activity was observed during exposure to white, green and blue lights (unpaired student’s *t*-test, two-tailed, p = 0.0021 for white treatment, p = 0.0005 for green treatment and p = 0.0010 for blue treatment; Figure 3), indicating significant differences between night and day activities and therefore sensitivity to these colours. For the no light daylight control and the red colour daylight, feeding activity during the daytime was not significantly different from the night activity (unpaired student’s *t*-test, two-tailed, p = 0.5125 for no light control, p = 0.0755 for red treatment; Figure 3 and Additional file 2).

### Opsin genes identification and analysis

**Figure 4:**
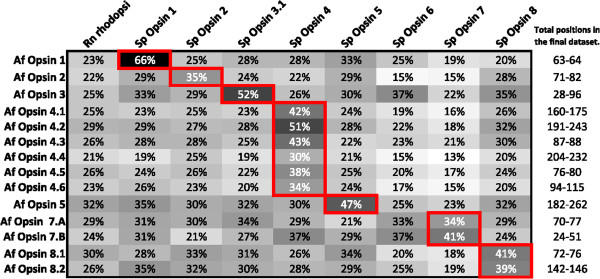
**Deduced amino acid sequences of Amphiura filiformis opsins (names in bold in the figure) aligned with Strongylocentrotus purpuratus opsins and Rattus norvegicus rhodopsin.** Alignment is limited to two highly conserved regions including the “DRY-type” tripeptide, the opsin-specific lysine residue, and the “NPxxY(x)_6_F” pattern. Predicted transmembrane alpha-helices are underlined in red. The lysine residue involved in the Schiff base formation – equivalent to K296 of the R. norvegicus rhodopsin - is highlighted in red in the alignment. The tyrosine residue (Y) in the position equivalent to the glutamate counterion E113 in R. norvegicus rhodopsin, and the DRY-type tripeptide motif (E134/R135/Y136 in R. norvegicus rhodopsin) is highlighted in blue. The pattern “NPxxY(x)_6_F” (position 302–313 of the R. norvegicus rhodopsin sequence) is highlighted in green. The amino acid triad (in the equivalent position 310–312 in the R. norvegicus rhodopsin) belong to the pattern NPxxY(x)6F. The “NxQ” motif, classically observed in c-opsins is written in red in the alignment and the “HxK” motif, classically observed in r-opsins, in blue. Other amino-acid residues that are highly conserved in the whole opsin family are shown with a grey background. See text and Additional file 3 for more details. Numbers indicated in gray on the left side of each aligned region correspond to the position number of the first amino acid of the considered sequence.

*In silico* analyses of the draft genome from *A. filiformis* revealed 14 opsin sequences. These opsins were named Af-opsin 1 [GenBank: KM276762], 2 [GenBank: KM276763], 3 [GenBank: KM276764], 4.1 [GenBank: KM276765], 4.2 [GenBank: KM276766], 4.3 [GenBank: KM276767], 4.4 [GenBank: KM276768], 4.5 [GenBank: KM276769], 4.6 [GenBank: KM276770], 5 [GenBank: KM276771], 7.A [GenBank: KM276772], 7.B [GenBank: KM276773], 8.1 [GenBank: KM276774], 8.2 [GenBank: KM276775], according to their similarity with the opsins of *S. purpuratus* (Sp-opsin 1, 2, 3.1, 3.2, 4, 5, 6, 7, 8) (Figure 2). These sequences were aligned with other known echinoderm sequences (Figure 4 and Additional file 3). Sequence analyses show conservative residues, which are represented in the alignment (Figure 4 and Additional file 3). BlastP results for the predicted protein, lengths of the gene fragments, as well as length and estimated molecular weight of the predicted protein sequences are shown in Additional file 4. The different analyses indicate 13 putative opsin genes as the sequences Af-opsin 7.A and 7.B do not overlap and therefore likely derive from a single gene (Af-opsin 7).

All sequences are characterised by the general structure of G protein-coupled receptors (GPCRs) comprising seven transmembrane (TM) domains (Figure 4 and Additional file 3). Numerous residues characteristic of opsins are present in the opsin sequences of *A. filiformis* (highlighted in Figure 4). However, as these sequences are incomplete, not all characteristic residues could be detected in all sequences. The Schiff base residue, a critical lysine required for covalent binding to the chromophore and considered as diagnostic for the opsin family, was detected in 9 opsin sequences from *A. filiformis*. The counterion is another opsin key functional residue, responsible for stabilising the inactive dark state pigment by helping to stabilise the protonated Schiff base and tuning the wavelength absorbance into the visible spectrum [61]. The ancestral opsin probably employed the negatively charged glutamate residue E181 (equivalent position in *R. norvegicus*) as the counterion [62]. E181 is indeed used as the counterion by diverse opsins such as peropsins, the cephalopod photoisomerase retinochrome and *Amphioxus* rhodopsin (Go-opsin) [54]. The majority of echinoid opsins and eight *A. filiformis* opsins (Af-opsin 1, Af-opsin 4.1, Af-opsin 4.2, Af-opsin 4.3, Af-opsin 4.4, Af-opsin 5, Af-opsin 7.A, Af-opsin 8.2) also present this E181 residue (Additional file 3). During the evolution of chordate opsins, the site of the counterion relocated to position 113 upon acquisition of a glutamate residue at that site (E113) (equivalent position in *R. norvegicus*) [61]. In most vertebrate visual opsins including *R. norvegicus* rhodopsin, the counterion is this glutamate residue E113 [53, 61]. In most invertebrate visual opsins, however, this residue is substituted by a neutral aromatic tyrosine Y [61, 62] that probably does not act as the counterion [63]. The majority of echinoderm opsins sequences, including four opsins of *A. filiformis* (Af-opsin 4.2, Af-opsin 4.4, Af-opsin 5, Af-opsin 7.B), seem to be characterised by an “invertebrate-type” tyrosine residue (Figure 4; see also [7]).

Other characteristic residues (also shared with GPCR family 1a,b,c members such as olfactory receptors, [64]) include the “DRY” tripeptide motif needed in the receptor transformation from an inactive to a G protein–coupled conformation [65]. Derived DRY-like tripeptide motifs are present in Af-opsin 1, Af-opsin 3, Af-opsin 4.2, Af-opsin 4.4, Af-opsin 5 and Af-opsin 7.B (Figure 4). Two non-contiguous cysteine residues needed for a possible disulfide bond involved in the stabilisation of the receptor are present in Af-opsin 4.1, Af-opsin 4.2, Af-opsin 4.4, Af-opsin 5, Af-opsin 8.1, Af-opsin 8.2 (Additional file 3). Another amino acid pattern, “NPxxY(x)_6_F” (position 302–313 of the *R. norvegicus* rhodopsin sequence), needed in G protein coupling like the DRY motif [65, 66], is observed in 9 of the 13 *A. filiformis* opsins (Af-opsin 2, Af-opsin 4.2, Af-opsin 4.4, Af-opsin 4.5, Af-opsin 4.6, Af-opsin 5, Af-opsin 8.1, Af-opsin 8.2; Figure 4). Within this pattern, an amino acid triad (in the equivalent position 310–312 in the *R. norvegicus* rhodopsin) is usually used to distinguish c- and r-opsins (NxQ in in the former and HxK in the latter, see [53, 65, 67]. The c-type NxQ motif was observed in Af-opsin 5 and Af-opsin 8.2 while the r-type HxK motif was found in Af-opsin 4.2, Af-opsin 4.4, Af-opsin 4.5 and Af-opsin 4.6 (Figure 4). Af-opsin 4.1 presents a derived motif HxS. Although the presence of these motifs is informative, it is certainly not sufficient to determine whether a sequence can be included or not in the ciliary/rhabdomeric opsin groups. Opsins from minor groups (Go opsins, neuropsins, peropsins, and basal-branch opsins) could indeed present the same motifs [53].

### Opsin gene expression

Three opsin mRNA sequences were retrieved from the Hi-Seq Illumina arm transcriptome of *A. filiformis* [Genbank: Biosample SAMN02934163]. Although these sequences are partial, their identification is unequivocal because they match perfectly the gene sequences of Af-opsin 2, Af-opsin 4.5, and Af-opsin 8.2. Alignments of mRNA and gene sequences (first translated into protein sequence) are presented in Additional file 5. Identity scores are superior to 99.9% for each of the three alignments.

### Opsin phylogeny

**Figure 5:**
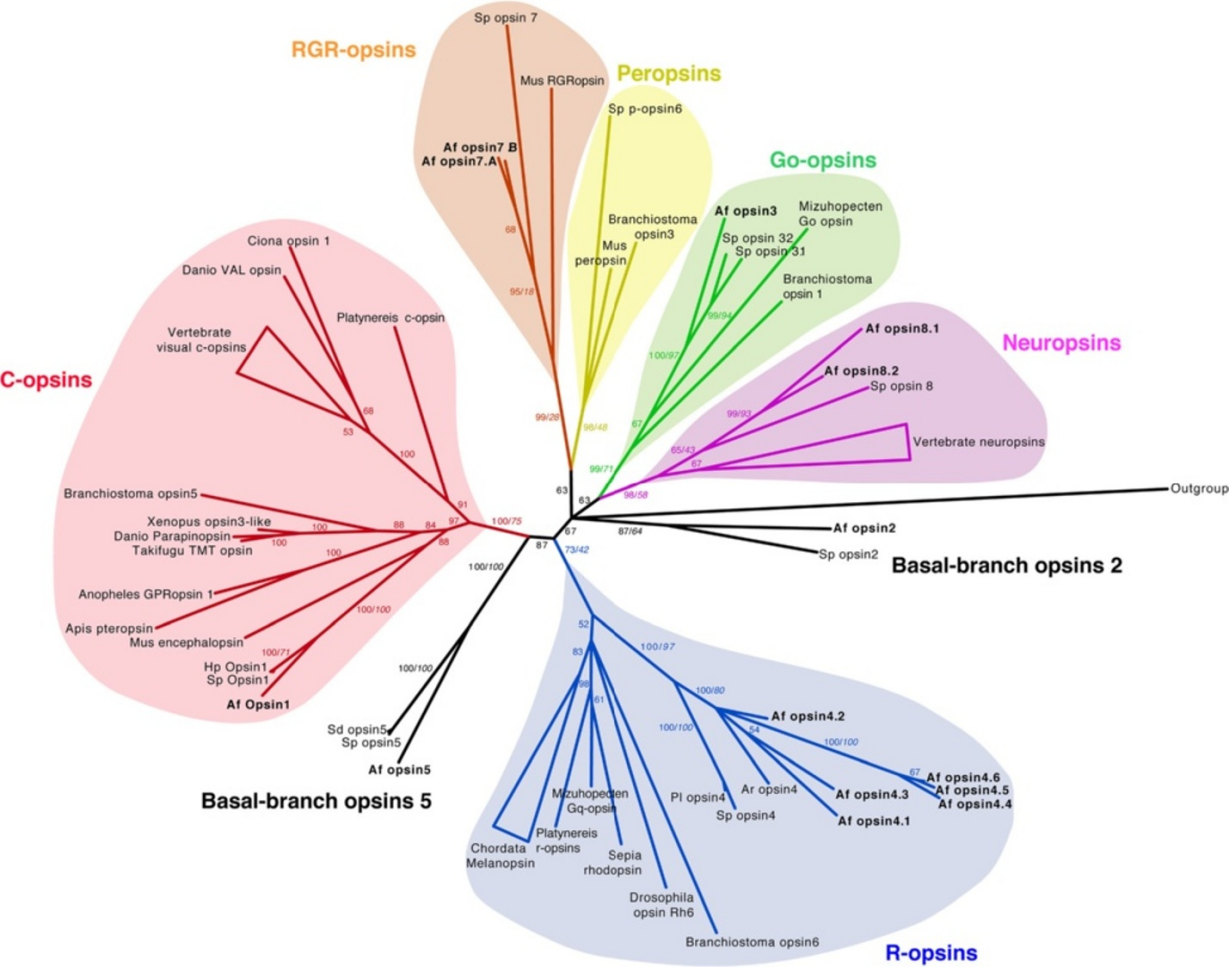
**Phylogenetic tree of metazoan opsins including the new opsins from Amphiura filiformis.** Representative bilaterian opsin members cluster into six significantly supported groups in both maximum likelihood and Bayesian analysis. Branch support values are indicated next to the branching points and correspond to posterior probabilities and boostrap proportions (in italics). Branch length scale bar indicate relative amount of amino acid changes (Bayesian analysis). A. filiformis opsins are represented in bold (Af). Other echinoderm opsins were included in the analyses: Strongylocentrotus purpuratus (Sp), Strongylocentrotus droebachiensis (Sd), Paracentrotus lividus (Pl), Hemicentrotus pulcherrimus (Hp), Asterias rubens (Ar).

The 14 new opsin sequences (9 *bona fide* opsin genes) and the 3 partial opsins mRNA (all first translated in protein sequence as described in the material and methods section) were included in the phylogenetic analysis together with other metazoan (including echinoderm) sequences representative of all opsin classes. The tree resulting from the Bayesian analysis is presented in Figure 5 and the maximum likelihood bootstrap proportion values are added to this tree (Additional files 6 and 7 present separate trees resulting from maximum likelihood and Bayesian analyses, respectively). The three main lineages are represented in the tree: the rhabdomeric lineage containing melanopsins and protostome visual opsins, the ciliary opsin lineage containing visual deuterostome opsins, encephalopsins and pteropsins and the “group 4” opsin lineage [53, 54] containing Go opsins, neuropsins, peropsins and RGR opsins. As several echinoderm opsins were included in this Group 4, the “exploded view” showing separately neuropsins, Go-coupled opsins, peropsins, and RGR opsins was chosen.

Echinoderm r-opsins are all clustered together and their common branch roots at the base of the rhabdomeric opsin group, close to the vertebrate melanopsins, supporting [4, 6, 7, 10, 26]. All *A. filiformis* r-opsin sequences are closely related to rhabdomeric opsins of other echinoderms and particularly the one of *Asterias rubens*, which is not surprizing considering that asteroids have been proposed to be the sister group of ophiuroids [68–71]. Three *A. filiformis* r-opsins (Af-opsin 4.4, Af-opsin 4.5, Af-opsin 4.6) are grouped together in a monophyletic clade that could indicate gene duplication in the lineage of ophiuroids. Af-opsin 1 is perfectly clustered with Sp-opsin 1. In the generated tree, these opsins and the c-opsin of the sea urchin *Hemicentrotus pulcherrimus* (Hp-opsin 1, [7]) branch basally to the subfamily of chordate c-opsins, including vertebrates rhodopsins and pinopsins as mentioned in [4, 10]. More specifically they are clustered with encephalopsins as previously observed in [4, 7, 26]. Af-opsin 2 grouped with Sp-opsin 2 in a basal position compared to the other opsin groups. In the same manner, Af-opsin 5 clusters with the *Sp*/*S.droebachiensis*-opsins 5 and has a basal position not included in a classical opsin group. Conversely to the purple sea urchin, *A. filiformis* is characterised by a unique Go-coupled opsin (Af-opsin 3). Af-opsin 7.A and 7.B are included with Sp-opsin 7 (not reported in the literature to the best of our knowledge) in the RGR opsin group. Considering our data, however, no peropsins were found in *A. filiformis*. Af-opsin 8.1 and Af-opsin 8.2 are clustered in the group of neuropsins and are closely related to Sp-opsin 8 (referenced as “opsin5-like”, not reported in the literature to the best of the authors’ knowledge).

### Opsin immunodetection

**Figure 6:**
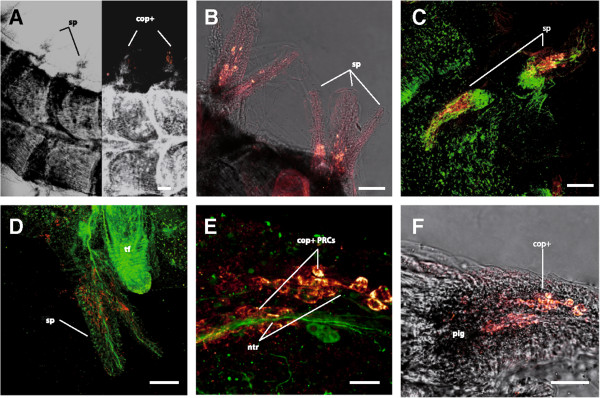
**Ciliary opsin positive cells in decalcified arms of Amphiura filiformis, detected by Confocal laser scanning microscopy.** Double immunolocalisation of c-opsin (red) and acetylated alpha-tubulin (green). **A**. Partially inverted transmission picture of an arm showing c-opsin positive cells/photoreceptor cells (cop + PRC) in the spines (sp). **B**. c-opsin proteins seem to be localised in the inner portion of the spines. **C**. Dense nerve tracts are present at the basis of the spines. **D**. Nerve tracts partially also run through an internal portion of the spines. Tube feet (tf) show no c-opsin positive cells. **E**. High magnification reveals connection of the c-opsin positive cells to the nerve tracts (ntr). **F**. Transmission view of a spine showing the internal c-opsin positive cells and the dark pigment (pig) at the spine base. Scale bars in **A-D**: 100 μm, **E**: 20 μm, **F**: 50 μm.

**Figure 7:**
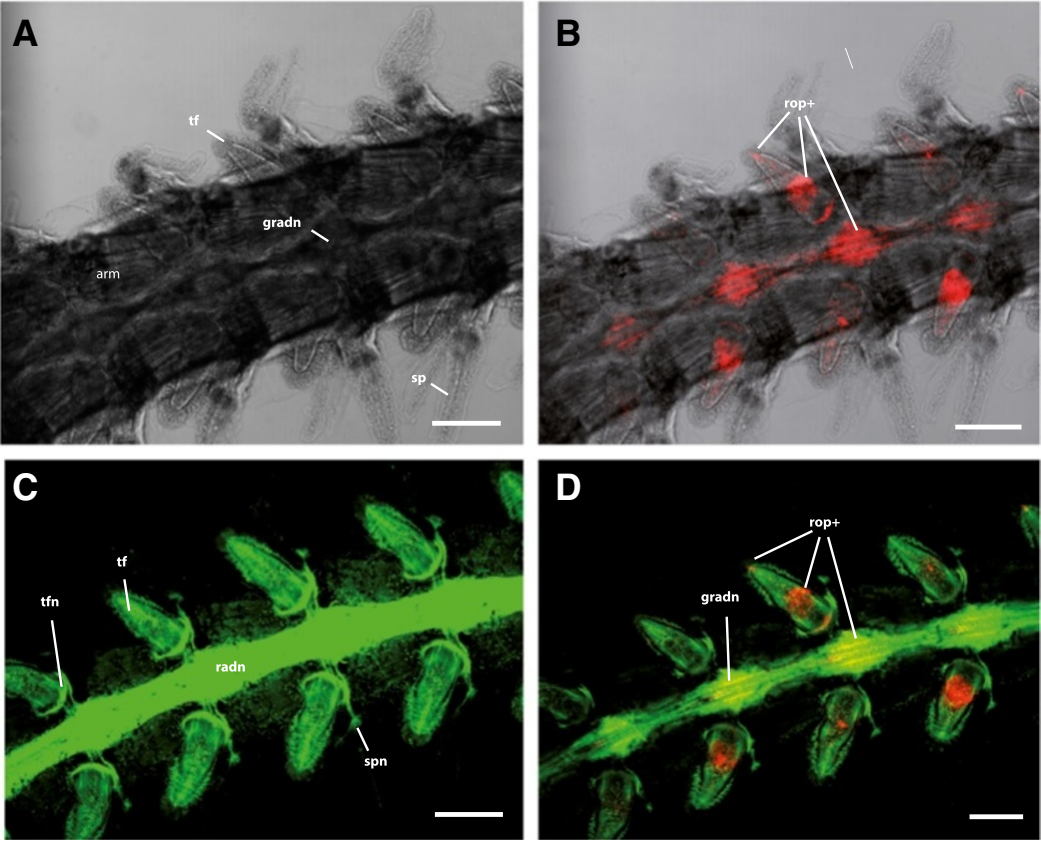
**Rhabdomeric opsin positive cells in decalcified arms of Amphiura filiformis, detected by confocal laser scanning microscopy.** Double immunolocalisation of r-opsin (red) and acetylated alpha-tubulin (green). **A**. Transmission view of arm (arm) with tube feet (tf) and spines (sp). **B**. R-opsin positive cells reside in tube feet and radial nerve. **C**. Innervation of arm showing connection of the radial nerve (radn) to each tube foot nerve ring (tfnr) and spine nerve (spn). **D**. Tube feet show r-opsin protein (rop+) presence within middle and tip region. R-opsins are also detected within radial nerves. R-opsin positive structures within the radial nerve likely represent axonal/dendritic projections and no stained cell bodies were observed. Scale bars in A-D: 200 μm.

The arms of *A. filiformis* consist of numerous articulated segments, each bearing a ventro-lateral pair of tube feet and three to six lateral pairs of spines (Figures 6A and 7A). In whole-mount preparations, the radial nerve is visible through the ventral plate on the midline of the arm and regular swellings are visible at the level of each tube foot pair (Figure 7A). Each tube foot is innervated by nerve fibres originating from the radial nerve and has a nerve ring surrounding its proximal part (Figure 7C). Another nerve strand connects the radial nerve to each spine.

Labelling with the anti-Sp-opsin 1 antibody revealed the localisation of c-opsins in the spines of *A. filiformis* (Figure 6B and C), confirming the observations of Ullrich-Lüter *et al.*[10]. Higher magnification views demonstrate the presence of these opsins in the inner part of the spines (Figure 6D). Nerve tracts, labelled with the anti-acetylated alpha-tubulin antibodies, clearly contact the c-opsin positive cells (Figure 6E). A dark pigmentation occurs at the base of the spines, in the immediate vicinity to the cells which are immunoreactive for c-opsins (Figure 6F).

**Figure 8:**
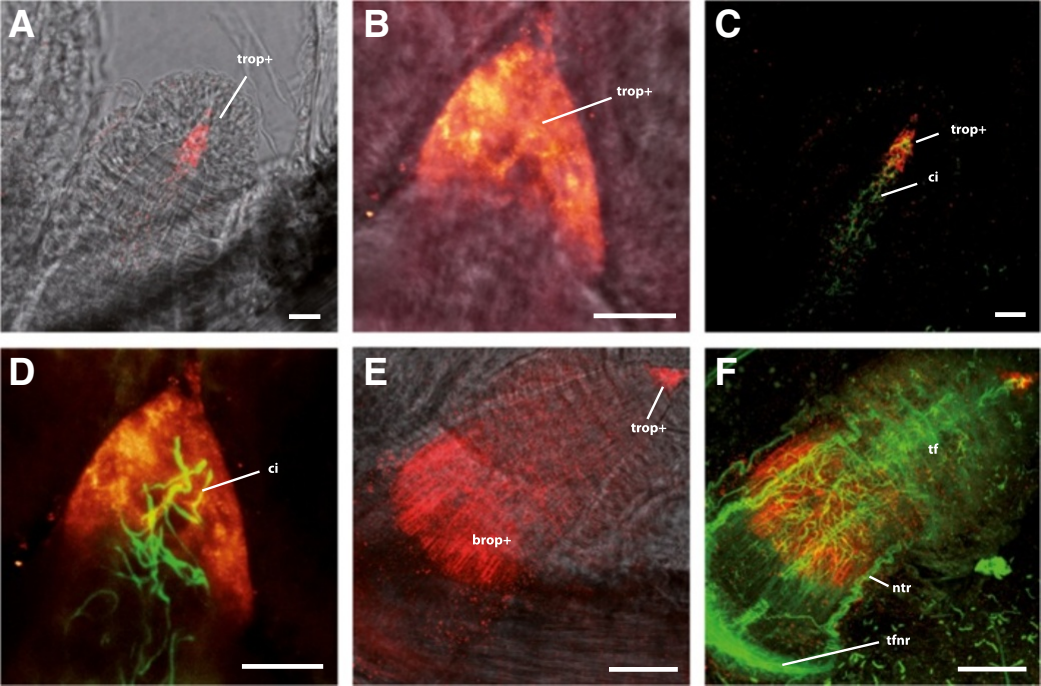
**Rhabdomeric opsin positive cells in the tube feet of Amphiura filiformis, detected by CLSM.** Double immunolocalisation of r-opsin (red/hot red) and acetylated alpha-tubulin (green). **A**. Transmission view of a tube foot (tf) with tip r-opsin positive cells (trop+). **B**. R-opsin staining shows a cone shaped morphology. **C**. Cilia (ci) are present along the longitudinal axis of the tube foot. **D**. Dark areas within the r-opsin positive region indicate location of nuclei. Cilia protrude from the r-opsin positive cells. **E**. Tube foot proximal r-opsin positive cells (brop+) showing a striated pattern. **F**. Dense nerve tracts (ntr) but also fine nerve fibers connect to the tube foot nerve ring (tfnr) in the area of proximal r-opsin positive cells. Scale bars in **A-D**: 10 μm, **E-F**: 50 μm.

Immunohistochemical labelling using anti-Sp-opsin 4 antibodies revealed r-opsin positive cells in the tube feet and within the swellings of the radial nerve (Figure 7B). In the former, immunoreactivity was observed in two distinct regions. The first immunolabelled region is cone-shaped and located at the most distal portion (tip) of the tube foot (Figure 8A). This cone shaped area, which presents a subepidermal localisation possibly corresponding to the nerve plexus, is homogeneously immunoreactive except for darker areas which presumably host the cell nuclei (Figure 8B). Cilia, visualised by the anti-acetylated alpha-tubulin antibodies, are present along the longitudinal axis of the whole tube foot (Figure 8C) and also emerge from the area labelled for r-opsins (Figure 8D). The second cluster of Sp-opsin 4-like positive cells is located in the middle portion of the foot and (Figure 8E), in contrast to the basal photoreceptor cell cluster of sea-urchin tube feet [9], it is clearly separated from the basal nerve ring (Figure 8F). At this level, the r-opsin positive cells are clearly located in the subepidermal nerve plexus and form a striped pattern contrasting the voluminous staining in the tip region. When images obtained with both anti-r-opsin and anti-acetylated-alpha-tubulin antibodies are merged, it can be observed that within the tip of the tube feet there is only a limited co-expression of the two proteins (Figure 8D), whereas in the basal tube foot portion both seem to be present in the same striated structures (Figure 8F). R-opsin positive structures were also detected in the arm radial nerve (Figure 7B).

## Discussion

### Light perception in *A. filiformis*

In ophiuroids, spectral sensitivity data are missing, especially for burrowing species such as *A. filiformis*. Rosenberg and Lundberg [40] showed that the activity pattern of this species is related to the photoperiodicity, the animals showing low or no activity at daytime and high activity at night. These authors assumed the existence of a “photoreception system” such as the one proposed by Hendler for *O. wendtii*[17, 31, 72]. However, these two species exhibit totally different behavioural patterns, with a change in diurnal activity in *A. filiformis* and a fast reaction upon shading in *O. wendtii*, making them difficult to compare in term of light perception. *A. filiformis* appeared to be sensitive to both green and blue light but not or weakly sensitive to red light (the p-value obtained for the red sensitivity test is close to the 0.05 threshold indicating a possible weak red light sensitivity). The purple sea urchin *S. purpuratus* reacts to light exposure by increasing tube foot and spine activities and rapidly moves away from the light source [9, 28, 73]. This species, therefore, shows a negative phototaxis upon illumination with a maximal reaction to the blue colour (450 nm), which is known to be the main colour present in the sea-environment [74]. However, *A. filiformis* was collected at 30–40 meters, in an environment where an ambient light shift to green has been observed, caused by the turbidity of the fjord and coastal waters [75]. A shift of sensitivity towards the green colour is, therefore, not surprising in this coastal water species.

Noteworthily, individuals of *A. filiformis* are bioluminescent and emit blue light [76, 77]. Although *A. filiformis* is apparently unable to perceive the bioluminescence from conspecifics [78], it can be hypothesised that it could use photoreception to perceive and control its own bioluminescence signal, as suggested for other species [79, 80]. Bioluminescence in brittle stars is indeed highly controlled (see [81] for review). This hypothesis is supported by the localisation of c-opsins in the spines of *A. filiformis* where bioluminescence occurs.

### Diversity of opsin genes in *A. filiformis*

The *in silico* analyses carried out in this study highlighted thirteen opsin genes in the ophiuroid *A. filiformis.* For nine of them, the *bona fide* opsin status was confirmed by, among other things, the presence of the Schiff base lysine. For comparison, the sea urchin genome contains nine opsins: one r-opsin, one c-opsin, two Go-opsins, one neuropsin, two basal-branch echinopsins, one peropsin and one RGR opsin. Even though the number of opsin genes is higher in the brittle star genome, opsin diversity (in terms of represented opsin classes) is higher in the sea-urchin genome with representatives in every classical opsin groups. Conversely, no peropsin was found in the available *A. filiformis* genomic data. However, Sp-peropsin (Sp-opsin 6) is not defined as a *bona fide* opsin because of the absence of the Schiff base residue and should be considered as a pseudo-opsin. The same number of *bona fide* opsin classes is therefore observed in both species, but gene repartition within the opsin classes differs. At some point, this gene repartition could be linked to the contrasted ecological differences between an epifaunal shallow water sea urchin and an infaunal deep-water brittle star.

The similarity search and phylogenetic analysis made it possible to ascribe *A. filiformis* sequences to the different opsin classes. No less than six r-opsins (Af-opsin 4.1 to 4.6), related to deuterostome melanopsins and protostome visual opsins, were identified. Homologues of “non-visual” rhabdomeric opsins (opsins 4) have been identified in many vertebrates [82, 83], in *Amphioxus*[84], and in echinoderms [4–6]. In vertebrates, opsins 4 (or melanopsins) are needed for non-image forming visual responses including the entrainment of the circadian clock to ambient light, light modulation of activity, and the pupillary light reflex [85–89]. Deuterostome animals are indeed generally thought to deploy ciliary type photoreceptors for vision [67, 90] even if multiple species have been shown to possess r-opsin expressing/rhabdomeric photoreceptors [91–97]. Within deuterostomes, sea stars seem to be the only known exception where r-opsin expressing photoreceptors are undoubtedly involved in image-forming vision [9, 18] even though it has also been strongly suggested for sea urchin rhabdomeric photoreceptors [9]. In this study, multiple duplication events in brittle star r-opsins would suggest a strong ecological importance of these opsins in the biology of these organisms. Light intensities reaching the depth where this brittle star lives are relatively low compared to terrestrial conditions for example. According to Fain *et al*. [98], rhabdomeric photoreceptors show an extremely high sensitivity and, in contrast to ciliary photoreceptors, are able to detect single photons.

A single c-opsin (Af-opsin 1) was highlighted in the genome of *A. filiformis* but the short size of the fragment made it impossible to confirm the presence of the Schiff base residue. However, blast results and the high similarity between the partial Af-Opsin 1 and Sp-Opsin 1 clearly indicate the homology between the two predicted protein sequences. One Go-coupled opsin was detected (Af-opsin 3), similar to Sp-opsin 3.1 and Sp-opsin 3.2. Homologue Go-coupled opsins are present in *Amphioxus*[99] but also in the ciliary photoreceptors of the scallop retina [100]. Two neuropsins (Af-opsin 8.1, Af-opsin 8.2) similar to Sp-opsin 8 (opsin 5-like) were also identified. Neuropsins (classically “Opsin 5” in vertebrates, but this number was already given to an “echinopsin” not included in the neuropsin group; see below) were recently identified in the human and mouse genomes [101–103] and are specific to deuterostomes. In mammals, neuropsins are expressed in the eye, brain, spinal cord and testis. In chicken and human, opsin 5 has been proposed as a UV sensor [85, 101–103]. Deep blue-UV receptors may have a biological relevance in marine species. Recently, UV sensitivity was highlighted in two species of anomuran crabs living at depths between 400 and 600 m [104]. The authors hypothesised that UV sensitivity might be related to bioluminescence perception. Opsins similar to Sp-opsin 2 and Sp-opsin 5 of *S. purpuratus* were identified in the *A. filiformis* genome. These two opsin types do not cluster in the classical opsin classes in our study confirming previous studies on *S. purpuratus* opsins [4, 10]. Moreover these two opsin types seem to be specific to the echinoderm lineage (echinopsins) and for that reason no conclusion about their function can be drawn. These two last opsins have a basal position in the phylogenetic opsin tree, which seems to indicate that these kinds of opsin were already present before the separation of the classical opsin groups. In this study, the opsin 5 group is present at the base of the ciliary opsin cluster and the opsin 2 group at the base of the rhabdomeric opsin cluster, as observed by Lesser *et al.* in [6].

The present study highlights the high diversity of opsin genes (c- and r-opsins, neuropsins, Go-coupled opsin, RGR opsin and basal-branch echinopsins) present in *A. filiformis*. Echinoderms thus appear to possess a large set of genes for light sensory capability comparable, in number, to the human opsin set (ciliary opsins – one rod opsin, three cone opsins, one encephalopsin/panopsin; rhabdomeric opsins – one melanopsin -, one neuropsin, one RGR-opsin and one peropsin; [102]). As a comparison, urochordates have at least 3 opsin genes [100] and cephalochordates at least 20 identified opsin genes [99].

### Opsin expression in *A. filiformis*

Among the 13 opsin genes of *A. filiformis*, only 3 were expressed in the arm transcriptome: one r-opsin (Af-opsin 4.5), one neuropsin (Af-opsin 8.2), and one basal-branch opsin (Af-opsin 2). The lack of detection of the other opsins could be a consequence of an expression pattern restricted to other life-history stages, tissues or environmental conditions, as well as of technical limitations such as the detection threshold of the Illumina transcriptome methodology. Alternatively, in case of low protein turnover, the lack of opsin mRNA is not necessarily correlated to the absence of the corresponding opsin protein. For example, the mRNA of Af-opsin 1, the c-opsin, is not present in the arm transcriptome, but the protein can be immunodetected in the arms of the brittle star using antibodies raised against Sp-opsin 1. However, in sea urchins, the expression of c-opsins was demonstrated to be 10-fold lower than the one of r-opsins [4, 5]. A similar expression pattern could explain the absence of c-opsin mRNA in the arm transcriptome of *A. filiformis*. The specific localisation of the c-opsin in the central part of calcified spines may also affect the quantity of opsin mRNAs that were obtained during extractions.

The presence of a c-opsin protein was observed in the spines of *A. filiformis* supporting [10]. C-opsin expressing photoreceptor cells were specifically located in the central tissues of the spines, which mainly comprise the axial nerve [105]. The co-localisation of acetylated-alpha-tubulin and c-opsin labelling indicates a close association between photoreceptor cells and the spine nervous system. In sea urchins, c-opsin (Sp-opsin 1) is expressed in pedicellariae but also in locomotory and buccal tube feet, spines and epidermis [10]. C-opsin was also detected in the aboral integument [26] and the spines [10] of the starfish *A. rubens*. Spines, therefore, appear to be a primary light perception system common to Echinoidea, Asteroidea and Ophiuroidea. However, Ullrich-Lüter *et al*. [10] showed that in *S. purpuratus*, *A. rubens* and *O. nigra* the c-opsin immunopositive photoreceptor cells are located in the spine epidermis, whereas in *A. filiformis* they are located deeper within the spine core tissues. One might wonder whether the spine ossicle could be involved in light focalisation as was proposed for the dorsal arm plates of the brittle star *O. wendtii*[31] and for the skeleton of sea urchin tube feet [9]. Pigments were observed in the basal part of the spines in *A. filiformis* close to the c-opsin positive photoreceptors, suggesting a relative directionality of light perception in these organs. In metazoans, photoreceptors are generally associated with pigments [106, 107] although it is actually not the case for the sea urchin tube foot photoreceptors [9]. As the spines of *A. filiformis* also constitute the photogenous areas that emit a blue light when the arms are stimulated [76, 77], ossicles and pigments could also be involved in light emission. Indeed, phenomena such as refraction, reflection, and transparency could even modify the direction of the composition of the light going into and/or out of the spine.

Immunostaining of the arms in *A. filiformis* using anti-Sp-opsin 4 antibodies showed the presence of r-opsins in the tube feet and in the radial nerve of the ophiuroid. In the former, immunolabelling was strong while it was much weaker in the latter. The immunodetected r-opsins are presumably orthologous to Sp-opsin 4. While it is not known whether the antibodies detect one or several r-opsins, the fact that the transcriptomic analysis reveals the expression of only one r-opsin mRNA in adult arms (on a total of 6 r-opsin genes detected in the genome) suggests that this opsin (Af-opsin 4.5) could be the only target of the anti-Sp-opsin 4 antibody.

In tube feet, two distinct regions present an extensive immunoreactivity, one at the tip, showing a cone-shaped morphology, and the second one in the basal area of the tube foot. This r-opsin distribution pattern is therefore similar to the one described in sea urchin tube feet [9] (for which only one r-opsin is described). R-opsins expressed in the tube feet of representatives species of Echinoidea [9] and now Ophiuroidea indicate that light sensitivity at the level of these appendages could be generalised in Echinodermata. Additionally, sea star optic cushions, known to arise from the ‘first primary podia’ [108], have rhabdomeric photoreceptors [23] expressing r-opsins [9]. Synaptid holothurian eyes, present on the feeding tentacles, are characterised by a microvillar photoreceptor structure and also derive from the ‘first primary podia’ [19]. The distribution of echinoid tube foot photoreceptors across the body was described as a derived “compound-eye” comparable to the classical rhabdomeric cerebral eyes of protostomes [9]. This analogy could be extended to ophiuroid tube foot photoreceptors. However, *A. filiformis* does not show any clear phototaxis or spatial vision and its tube foot photoreceptors are presumably involved in non-visual photoreception. Due to their high sensitivity [98], rhabdomeric photoreceptors would have a functional advantage for differentiating diurnal cycles under the ambient dim light conditions found in deeper water. The detected r-opsin positive cells in the tube feet of *A. filiformis* are potential candidate photoreceptor cells mediating the photoactivity pattern described by Rosenberg and Lundberg [40]. Moreover, the photoreceptors needed for ambient light perception would not rely on a shading device such as pigments and, conversely to spines, pigments were not observed in tube feet. Rhabdomeric photoreceptors in *A. filiformis* would thus be able to perceive the light but without being able to determine the direction of light.

R-opsin immunoreactivity was also highlighted within the radial nerve cord. R-opsins could be expressed in cells closely related to the nerve cells but nerve cells could also be directly photosensitive as it was proposed for some echinoderm species [12–14, 17, 27, 28, 109–113] or other invertebrates [27, 114, 115]. Considering the co-localisation of anti r-opsin and anti-acetylated-alpha-tubulin antibody staining in the radial nerve cord, this second hypothesis is proposed. The radial nerve cord localisation is the only one that might be associated with the “global light receptive system” proposed by Hendler [12, 31, 72] for some ophiuroids. Analyses of z-stacks indicate the presence of the r-opsin-like labelling within a central portion of the radial nerve cord that would thus not coincide with the localisation of the presumed photoreceptor cells of *O. wendtii*. According to Hendler’s model, the photoreceptors of *O. wendtii* would have to be present in a nerve bundle located on the focal plane of the microlenses below the dorsal arm plates [12, 31], and would therefore be clearly aboral and distinct from the oral radial nerve [116]. Moreover, unlike *O. wendtii*, *A. filiformis* does not show any lens-like structure at the level of the dorsal plates (J.D., personal observations). The different lifestyle of *A. filiformis* in comparison with *O. wendtii*, however, does not exclude that these photoreceptors might be deployed in association with other arm ossicles.

## Conclusion

The present study highlights the large diversity of opsin genes detected in the brittle star *A. filiformis*, with thirteen putative opsin genes distributed among ciliary and rhabdomeric opsins, Go-coupled opsins, neuropsins, RGR opsin and “echinopsins” (basal-branch opsins specific to echinoderms). Considering the derived “non-visual based ecology” of this burrowing brittle star, this important light perception toolkit is surprising. Based on immunodetections and expression data, it is proposed that this brittle star species exhibits an opsin-based photoreception system mediated mainly by two opsins, one ciliary and one rhabdomeric, in the adult arms. R-opsins, mainly expressed in tube feet, might be linked to ambient light perception needed for the synchronisation of the feeding activity to the nycthemeral cycle. C-opsins, only expressed in spines, could be involved in the bioluminescence control process.

## Availability of supporting data

The Illumina derived short read files (*A. filiformis* adult tissues) are available at the NCBI Sequence Read Archive (SRA) under the study accession number SRR1523743 [117]. Truncated opsin alignment used for phylogenetic analyses and ML and Bayesian tree files were uploaded on the DRYAD repository (http://www.datadryad.org) [118–120].

## Electronic supplementary material

Additional file 1: **List of the reference opsins used for blast (A) and phylogenetic analyses (A,B).** (XLSX 45 KB)

Additional file 2: **Statistical analysis of the data from the behavioural experiments.** (XLSX 12 KB)

Additional file 3: **Deduced amino acid sequences of** ***A. filiformis*** **opsins (names in bold in the figure) aligned with Strongylocentrotus purpuratus opsins and Rattus norvegicus rhodopsin.** Only the “TM cores” of the opsins are aligned. N-terminus and C-terminus ends are written in light gray. Predicted transmembrane alpha-helices are underlined in red. The Schiff base residue – equivalent to the lysine residue in the position 296 of the R. norvegicus rhodopsin - is highlighted in red in the alignment. Two cysteine residues potentially involved in a disulfide bond are highlighted in yellow (positions equivalent to C110 and C187 in R. norvegicus rhodopsin, present after the II TM and the IV TM). A potential palmitoylation motif composed of two contiguous cysteine residues (positions equivalent to C322 and C323 in R. norvegicus rhodopsin) is also highlighted in yellow at the C-terminus. The tyrosine residue (Y) in position equivalent to the glutamate counterion E113 in R. norvegicus rhodopsin, glutamate counterion candidate E181 and DRY-type tripeptide motif (E134/R135/Y136 in R. norvegicus rhodopsin) present at the top of the III TM ([63 95]) is highlighted in blue. The pattern “NPxxY(x)_6_F” (position 302–313 of the R. norvegicus rhodopsin sequence) is highlighted in green. The amino acid triad (in the equivalent position 310–312 in the R. norvegicus rhodopsin) belong to the pattern NPxxY(x)6F. The “NxQ” motif, classically observed in c-opsins, is written in red in the alignment and the “HxK” motif, classically observed in r-opsins, in blue [52 61]. Other amino-acid residues that are highly conserved in the whole opsin family are shown with a gray background [52, 59]. See text and the legend of Figure 4 for more details. Alignment edited in strap software (http://www.bioinformatics.org/strap/). (PDF 156 KB)

Additional file 4: **BLASTP results and characteristics of the** ***A. filiformis*** **opsin genes.** (XLSX 55 KB)

Additional file 5: **Alignments of** ***A. filiformis*** **opsin mRNAs and corresponding genes first translated in protein sequences.** The alignment sizes are 33, 32 and 33 amino acids for Af-Opsin 2, Af-Opsin 4.5 and Af-Opsin 8.2 alignments, respectively. (PDF 102 KB)

Additional file 6: **Phylogenetic tree of metazoan opsins, including the new opsins from Amphiura filiformis, obtained using maximum likelihood inference.** Branch length scale bar indicate relative amount of amino acid changes. Branch support values, corresponding to bootstrap proportions, are shown next to the branching points. A. filiformis opsins are represented in bold (Af). Other echinoderm opsins were included in the analyses: Strongylocentrotus purpuratus (Sp), Strongylocentrotus droebachiensis (Sd), Paracentrotus lividus (Pl), Hemicentrotus pulcherrimus (Hp), Asterias rubens (Ar). (PDF 416 KB)

Additional file 7: **Phylogenetic tree of metazoan opsins, including the new opsins from Amphiura filiformis, obtained using Bayesian inference.** Branch length scale bar indicate relative amount of amino acid changes. Branch support values, corresponding to Bayesian posterior probability, are shown next to the branching points. A. filiformis opsins are represented in bold (Af). Other echinoderm opsins were included in the analyses: Strongylocentrotus purpuratus (Sp), Strongylocentrotus droebachiensis (Sd), Paracentrotus lividus (Pl), Hemicentrotus pulcherrimus (Hp), Asterias rubens (Ar). (PDF 216 KB)
