# Supplementary material for: High opsin diversity in a non-visual infaunal brittle star
Source: BMC Genomics. 2014 Nov 28;15:1035. doi: 10.1186/1471-2164-15-1035 (PMC4289182; doi:10.1186/1471-2164-15-1035)
Supplement: Supplementary file 3 — Additional file 3: Deduced amino acid sequences of A. filiformis opsins (names in bold in the figure) aligned with Strongylocentrotus purpuratus opsins and Rattus norvegicus rhodopsin. Only the “TM cores” of the opsins are aligned. N-terminus and C-terminus ends are written in light gray. Predicted transmembrane alpha-helices are underlined in red. The Schiff base residue – equivalent to the lysine residue in the position 296 of the R. norvegicus rhodopsin - is highlighted in red in the alignment. Two cysteine residues potentially involved in a disulfide bond are highlighted in yellow (positions equivalent to C110 and C187 in R. norvegicus rhodopsin, present after the II TM and the IV TM). A potential palmitoylation motif composed of two contiguous cysteine residues (positions equivalent to C322 and C323 in R. norvegicus rhodopsin) is also highlighted in yellow at the C-terminus. The tyrosine residue (Y) in position equivalent to the glutamate counterion E113 in R. norvegicus rhodopsin, glutamate counterion candidate E181 and DRY-type tripeptide motif (E134/R135/Y136 in R. norvegicus rhodopsin) present at the top of the III TM ([63 95]) is highlighted in blue. The pattern “NPxxY(x)6F” (position 302–313 of the R. norvegicus rhodopsin sequence) is highlighted in green. The amino acid triad (in the equivalent position 310–312 in the R. norvegicus rhodopsin) belong to the pattern NPxxY(x)6F. The “NxQ” motif, classically observed in c-opsins, is written in red in the alignment and the “HxK” motif, classically observed in r-opsins, in blue [52 61]. Other amino-acid residues that are highly conserved in the whole opsin family are shown with a gray background [52, 59]. See text and the legend of Figure 4 for more details. Alignment edited in strap software (http://www.bioinformatics.org/strap/). (PDF 156 KB) [file 12864_2014_6862_MOESM3_ESM.pdf]

```
Rn Rhodopsin   1    MNGTEGPNFYVPFSNITGVVRSPFEQPQYYLAEPWQFSMLAAYMFLLIVLGFPINFLTLYVTVQHKK.LRTPLNYILLNLAVADLFMVFGGFTTTLYTSLHGYFVFGPTGCNLEGFFAT
Sp Opsin 1     1    MNYSTPVMTSTASVSGPSPWTSTLESKAMSNLMTGLVTNVNALSGIGNETPTTIGLSSLVVPVSRTTYNYLTVYTGFLTIFGILNNGIVMILFARFPS.LRHPINSFLFNVSLSDLIISCLASPFTFASNFAGRWLFGDLGCTLYAFLVF
Sp Opsin 2     1    ......MT
Sp Opsin 3.1   1    MAASVTESSATEAISRLEPEYMVPLTRTGYLLTAIYLTIVGSIATVGNITVICVLCRYRTFRKRSINLLLINMAASDLGVSVAGYPLTTVSGYWGRWLFGDVGCQFYAFCVY
Sp Opsin 4     1    MNAVTTALPHGLNKPTIEARWTKSLRTPPNMLIVNLAISDFGMVITNFPLMFASTIYNRWLFGDAGCQFYAFCGA
Sp Opsin 5     1    MPTTLMENSTPGWMADDSQMEETHPAFPLIGGYLLVVVLLGTAGNSLVIYTFLRFKK.LHSPINLLIVNLSASDLLVATTGTPLSMVSSFYGRWLFGTNACAFYGFVNY
Sp P-Opsin 6   1    ......MAICCIPLSTTANFIQRWPFHWAGCKFYGFFFM
Sp Opsin 7     1    ......MSPVVAVSSFSEEWVYGSSGCQTYGFVAN
Sp Opsin 8     1    MDVNAKWWTNETLRTRDQFSDDHYTSVLSYEGDIWAGVYLMFISLIAFIGNISVIVISLRKREKLK.PIDLLTINLAIADFLICVVSYPLPMISAFRHRWSFGKFGCVWYGFTSF
Af Opsin 1     0    ......
Af Opsin 2     0    ......
Af Opsin 3     1    ......IIATICNVIVILVLLKKNTFKKRSVNILLLNIACSDLAISFSGYPLFTASNYAGRWIAGVAGCKIAGFTVY
Af Opsin 4.1   0    ......
Af Opsin 4.2   1    ......TRSLRTPPNMLIINLACSDLLMVFFEFPMMFLSTVHGRWLFGEVGCDAYAWGGA
Af Opsin 4.3   0    ......
Af Opsin 4.4   1    ......IVLCVCVSVLIFLVFVWSFYHVYHCINLYLYLHF
Af Opsin 4.5   0    ......
Af Opsin 4.6   0    ......
Af Opsin 5     1    MENGTYTLSTNTHHDYESMMSSQDVFDKIYFPGKTAVGFYLAFVSLFGTVGNAIVILLIYKTST.LRTPTYILILNLSVSDLIVSCFGAPMSCTSSFVGRWLYGNIGCNIYGFINY
Af Opsin 7.A   0    ......
Af Opsin 7.B   1    ......MCPFAASASFTESWPFGETGCQTYAFFGM
Af Opsin 8.1   0    ......
Af Opsin 8.2   0    ......

Rn Rhodopsin   119  LGGEIGLWSLVVLAIERYVVVCK.PMSNFRFGENHAIMGVAFTWVMALACAAPPL..VGWSRYIPE.GMQCSCGIDYYTLKPEVNNESFVIYMFVVHFTIPMIVIFFCYGQLVFTVKEAAAQQQESAT......
Sp Opsin 1     150  VAGTEQIVILAALSIQRCMLVVR.PFTAQKMTHRWALFFISLTWIYSLIICVPPL..FGWNRYTYE.GPGTACSVAWNS..PSPGDTSYIIFIFVLVLVIPFGIIIFCYGLLVYAVKKISRTQAALSS......
Sp-Opsin 2     3    FLGLNSLMSHAVIAVDRYLVITK.PHFGIVVTYPKAFLMISIPWVFSFAWAVFPL..AGWGEFTYE.GTGAWCSVRWDS..DQPQIMSYVLAMMFLTFISSIVIMMYCYICIFLTTRRMPRWATSNSI......
Sp-Opsin 3.1  113  TLSCSTISTHAAIAVYRYIYIVK.TDLRPKLTANFTSGVIVVIWVYAFFWTVTPF..VGWSSYIYE.PFGTSCSVNWVG..RTISDISYMVACTIGVYLLQIFIMLYCYIRVAKKIRGVDPGRTEEKD......
Sp Opsin 4     76   LFGIMSIANMTAIALDRYYVICWSLEAVRSVTHRRSMIIIIIVWCYAIFWSIPPF..FGVGSYVLE.GYGLGCTFDFMT..KDLNHYLHVSFLFASSFVVPVTIIIVCFTRIAITVRAHRHELNKMRT......
Sp Opsin 5     109  YCGCISLNSLAAISVFRYIIVVRGQAQNNKLSLRSSIYAILVIHLYTLIFSTPPL..YGWNRFVLA.GYHTSCDIDFHT..KTPLFVSYICYMFFFLFFLPLGLISWSYFKIYQRVSKHSNSMRTSFTGVTKEINSDEKHAWLEKMKTTQ
Sp P-Opsin 6   34   FFGLTTVGNTVTLAVSRYLIVCR.SELAQQLTFSHYRYFAMSAWVNGLFWFAALMPIFGWSRYDIDSPLQTSCFVDWQR..IDLSYVSYIVSWFIINFVLPLSLMVFCYVSAFLTRQEEAGEAEEGQF......
Sp Opsin 7          FFGLISIWSLVAMVLHHY.QSSK.IGAKRDDISSRYSMTIALIWGGAFFWSATPLPFIGVGRYVVE.PFGTGCLLDFAD..RSPSYFIYLVGFSTLGLAFPIALLISRGLNYEKVPI......
Sp Opsin 8     115  LFAVGSMATLMVIALLRYAKLCR.ENVDQYQSRPFVIKVIVAIWGFAFFTTAPPL..FGWSSYVPE.PYHLSCTIDFAD..TSPSGLSYTYFTTIVVFFMPLMIIVLCYVAIARKMIHHNRRINVGHN......
Af Opsin 1     1    ...CEQIVALAAVSMQRCFLVVR.PFTARKMTSSWAFLALVITWIYSLTISLPPA..FGWNDYVIE.GAGT......
Af Opsin 2     0    ......
Af Opsin 3     72   FFSSVTIVTYAYIAYYRYIYVCK.PNT......
Af Opsin 4.1   1    ......CYSLEKLNSISYHRAFAMVGLVWIYSFIWAILPF..VGIGEYVLE.GYHVSCTFQYLN..QSIRNKIYVGCLYCGAFVFPVSVIAICYWKMYQKVKATRKTLISAVA.....GMTGIHRGGCSGKNQS
Af Opsin 4.2   55   MFGVLSISTLTAIAFDRQYAICSSLDKLRNITYGRAGRMVVCVWLYSVFWSIFPF..FGIGDYVLE.GYGVSCTFHYLD..TSRRNRIYVGFLFIGDFFLPLCAIISCYVHIVGTVRANRKNLADISK......
Af Opsin 4.3   1    ......HYV.ICSGSYLVRRVSYYRWLIMAMVVWSYSFFWAILPL..FGIGKYVHE.GYGVSCSFEYLD..VSRHNRLYVGFLFVGGFLIPVSIITVCYSRIVQRVHSSRRALSGSCPSNTYLIKRKTEIK......
Af Opsin 4.4   40   VF........CKLYLYRRCAICTTTDGSGTGSYRRVFFLTVVAWVYSFLWSILPL..SGKGAFVLE.GYKLNCSFDYVT..QTLENKLYVGFLFAGAFFIPMTVIMYSYTRVVLAVKQSRQSLLDMSK......
Af Opsin 4.5   1    ......MGKSTAGVT......
Af Opsin 4.6   1    ......IPMIIIIYSYTRVVLAVRKSRQTFLDMGK......
Af Opsin 5     116  YCGCISLNSYAVIAIVRYLKVVR.RSVGSTILKTHVVRVIYAVHVYTFIFTIPPL..FGWNDFVLE.GFNTQCDIAYKI..KTPLYISYVSVIFIALFFIPLFIITFCYVRIVQYVSKHGTRLRKSMN......
Af Opsin 7.A   1    ......HPAKSSSLLVMAIWVNALFWGVAPLNPIRWGRYTVE.PFGTGCLLDFES..RDIMYLAYLLVMVVVCFVIPVGAMIYCAINVK......
Af Opsin 7.B   30   TFGIASVTNLAALTGDIYHETQ......
Af Opsin 8.1   0    ......
Af Opsin 8.2   1    ......LWVYSFIIAILPI..LGISSYTTE.PYNISCTINWTG..SSLEDKMYVILSVVFGFGIPLLICITCYVLIFKQLTCRNNAIRAPRH......

Rn Rhodopsin   243  ......TQKAEKEVTRMVIIMVIFFLICWLPYASVAMYIFTHQGSNFGPIFMTLPAFFAKTASIYNPIIYIMMNKQFRNCMLTTL...CCGKNPLGDDEASATASKTETSQVAPA
Sp Opsin 1     272  ......EAKADRKVSKMIFIMILFFLIAWTPYTGFSLYVTFGKNVVITPLAGTFPPFFAKLCTIHNPIIYFLLNKQFKDALIQLF...CCGENPFDRDESEHEGRGGRHRHRTAPSATAHIGGRGRAS(X)100
Sp Opsin 2     125  ......KTHERNRRRREQKLLKTLIAIAIAFLVAWSPYAITSMIVVFGGSELLSLTATTLPSLFAKSSVMINPIIYAVTSRVFRKSLKKMLTSFFPGCMTYIMTDKSPPSSSRPIQLGLSSDSKLKKDDQTSSC(X)224
Sp Opsin 3.1   235  ......AGVVVFGRLRKREAKIDTHVTKMCFMMMLTFIVVWAPYAVECLRAAHV..HRISALSSVLPTMFAKSSCMVNPIIFLTSSSKFRQDLGKLWSRPSSQDSLQLEERNKTQRSLYVRHSELGSAHGNDTASVYYEK(X)131
Sp Opsin 4     199  .....KLTEDKDKKHKSSIRRANKAKTEFQIAKVGFQVTIFYVLSWMPYSIVAVIGQYFDSDLLTPLGTVVPVIFAKCSAIWNPIIYCLSHEKFNAALKEKLMGMCGIEIPSKHRSMGSQESSVTGRRGMHRQNSSTLSESSVTS(X)100
Sp Opsin 5     256  ILHKPVTFLRLKSSFEPRFKPRFKRRFNHRRTASTLFVTIVVFLFAWFPYCIVSLWVLIGDANSISKLSTTIPSLFAKSSVIYNPLIYVVLNSKFRKALIQTLSFLKCLSKHELSESS
Sp P-Opsin 6   157  ........AADPTRNDEPSPNDVDWASQPEAHWIGIVTVVVFVLSWVPYSVLLLYVISNDPTEMPTYLPMVAPLFAEITLWIHPILFLVCVKKFRSYAIMMI...CCRTEVEAIEVDPQANDSHRMSEARRFADHFV
Sp Opsin 7     142  ......ESVIACWKAVLVLCFYWGCYGLVAIATALSGPGRVSVRLFAIAPLLAKTCPIVNAVIFGDTMSLDEPTTTKEQKKH
Sp Opsin 8     237  ......AGRML..........LEIRLLKTACMITMAYTISWTPYAVIAMWVTYIPVNQIPDAFRILPAFCAKTSSVYNPIIYCIFNKSFRQDLSSLI...CCCACQCYTITINLDINSHAQQQFRRIEERRDEVGTYKRR(X)230
Af Opsin 1     /    ......
Af Opsin 2     1    ......DVSRRLQLHSTDEEQKKFKRERKVLRVMMSISLTYFAAWTPYGILGLWATFGDPSQIPLFLITSGSMCGKASTALNPLIYTSTNRAFRGSIKK......
Af Opsin 3     /    ......
Af Opsin 4.1   121  KGVSMRGVGKYTTSKRKSIDDRWRGQKLELQIARVGAFLTILFVVSWTPYATVALIGQYINPDLVTPLSQTIPVVFAKCSAAWDPFVYAIKHTSFRSALHAQFGKKTRQRVRGSNKENDSKAKLDAAGERSSSKSKHLNRDEGELY(X)4
Af Opsin 4.2   178  ......DESIKKDKKGKKKSKRQNSEYAIAKIGMTLTALFALSWTPYATVAFIGEYINGDLLGPMVQTLPVVLAKSSAIWNPIVYAISHKKFKAAIRDHFIKKCCGELPETNFQSQGTSRDSLSSADTLRQSSKKSSHHKHAP(X)85
Af Opsin 4.3   /    ......
Af Opsin 4.4   155  ......VKTTLVVRTFKIHKRQVKNFETAKIGMKLITMFVLSWGPYACVAFIGQFVSPTLVFPIIQLIPVVMAKSASVWNPIVYAISHRRFKRELRGIFLEKCCQGMPTVDETSATGSYTGEFFNDFNDQ
Af Opsin 4.5   10   ......SFRKHKRKVKNYETAKIGMKLISLFVLSWGPYASVAFIGQFVNPSLMFPLLQLIPVVMAKSASVWNPMVYAISHRRFKRQLRTIFLEMFCAGVSMAETTSQISSNSNAARFKEFRVHSKVEAAAV
Af Opsin 4.6   30   ......STAGVTSFRKHKRQVKNYETAKIGMKLIALFVLSWGPYASVTFIGQFVNPALMFPLLQLIPVVMAKSASVWNPMVYAISHRRFKRQLRAIFLEMFCAGVSLAETTSQISSN..AARFKKFRAHSKAEAAAV
Af Opsin 5     238  ......RIRSRRESIGSSSKTTLMVLICIIVYLVTWLPYCIVAFWALFGDPSAISPPMSAAPALLAKASSIFNPWIFAGLNSQFRRALKV......
Af Opsin 7.A   /    ......
Af Opsin 7.B   /    ......
Af Opsin 8.1   1    ......SSFAMTICFIILWMPYAVVTLWNAFHGDNSVPLWATAIPVVIAKSSSLFNPTIYLIYNKSFRKDTNEII..QCCGCRCCCVAVNENPNNWRRVANCRELDVYGEPGIGSTTQ
Af Opsin 8.2   82   ......MESRLVKTAFAVMLCFMLAWTPYSLVSVWSTIQGEHTLPMWASVIPVLCAKSSTVFNPVIYMVFNKQFREDVTTLFY..CCGCRCYMFSIHTDTSEWDNAVKGVLQGKRYNIYDISCKI(X)121
```
