# Supplementary material for: High opsin diversity in a non-visual infaunal brittle star
Source: BMC Genomics. 2014 Nov 28;15:1035. doi: 10.1186/1471-2164-15-1035 (PMC4289182; doi:10.1186/1471-2164-15-1035)
Supplement: Supplementary file 5 — Additional file 5: Alignments of A. filiformis opsin mRNAs and corresponding genes first translated in protein sequences. The alignment sizes are 33, 32 and 33 amino acids for Af-Opsin 2, Af-Opsin 4.5 and Af-Opsin 8.2 alignments, respectively. (PDF 102 KB) [file 12864_2014_6862_MOESM5_ESM.pdf]

Af-Opsin 2

```
                                          1
Af Opsin 2 - Draft Genome(KM276763)       DEEQKKFKRERKVLRVMMSI SLTYFAAWTPYGILGLWATF
Af Opsin 2 - Illumina(SRR1523743)         -------------------- --------------------

                                          41
Af Opsin 2 - Draft Genome(KM276763)       GDPSQIPLFLITSGSMCGKA STALNPLIYTSTNRAFRGSI
Af Opsin 2 - Illumina(SRR1523743)         -DPSQIPLFLITSGSMCGKA STALNPLIYTSTNR------

                                          81
Af Opsin 2 - Draft Genome(KM276763)       KK
Af Opsin 2 - Illumina(SRR1523743)         --
```

Af-Opsin 4.5

```
                                          1
Af Opsin 4.5 - Illumina(SRR1523743)       -------------------- ---------GPYASVAFIGQ
Af Opsin 4.5 - DraftGenome(KM276769)      VRKSHKRKVKNYETAKIGMK LISLFVLSWGPYASVAFIGQ

                                          41
Af Opsin 4.5 - Illumina(SRR1523743)       FVNPALMFPLLQLIPVVMAK S-------------------
Af Opsin 4.5 - DraftGenome(KM276769)      FVNPSLMFPLLQLIPVVMAK SASVWNPMVYAISHRRFKRQ

                                          81
Af Opsin 4.5 - Illumina(SRR1523743)       ------------
Af Opsin 4.5 - DraftGenome(KM276769)      LRTIFLEMFCAG
```

Af-Opsin 8.2

```
                                          1
Af Opsin 8.2 - DraftGenome(KM276775)      MLCFMLAWTPYSLVSVWSTI QGEHTLPMWASVIPVLCAKS
Af Opsin 8.2 - Illumina(SRR1523743)       -------------------- --------WATVIPVLCAKS

                                          41
Af Opsin 8.2 - DraftGenome(KM276775)      STVFNPVIYMVFNKQFREDV TTLFYCCGCRC
Af Opsin 8.2 - Illumina(SRR1523743)       STVFNPVIYMVFNKQFREDV T----------
```
