# Supplementary figures and images for: High opsin diversity in a non-visual infaunal brittle star
Source: BMC Genomics. 2014 Nov 28;15:1035. doi: 10.1186/1471-2164-15-1035 (PMC4289182; doi:10.1186/1471-2164-15-1035)

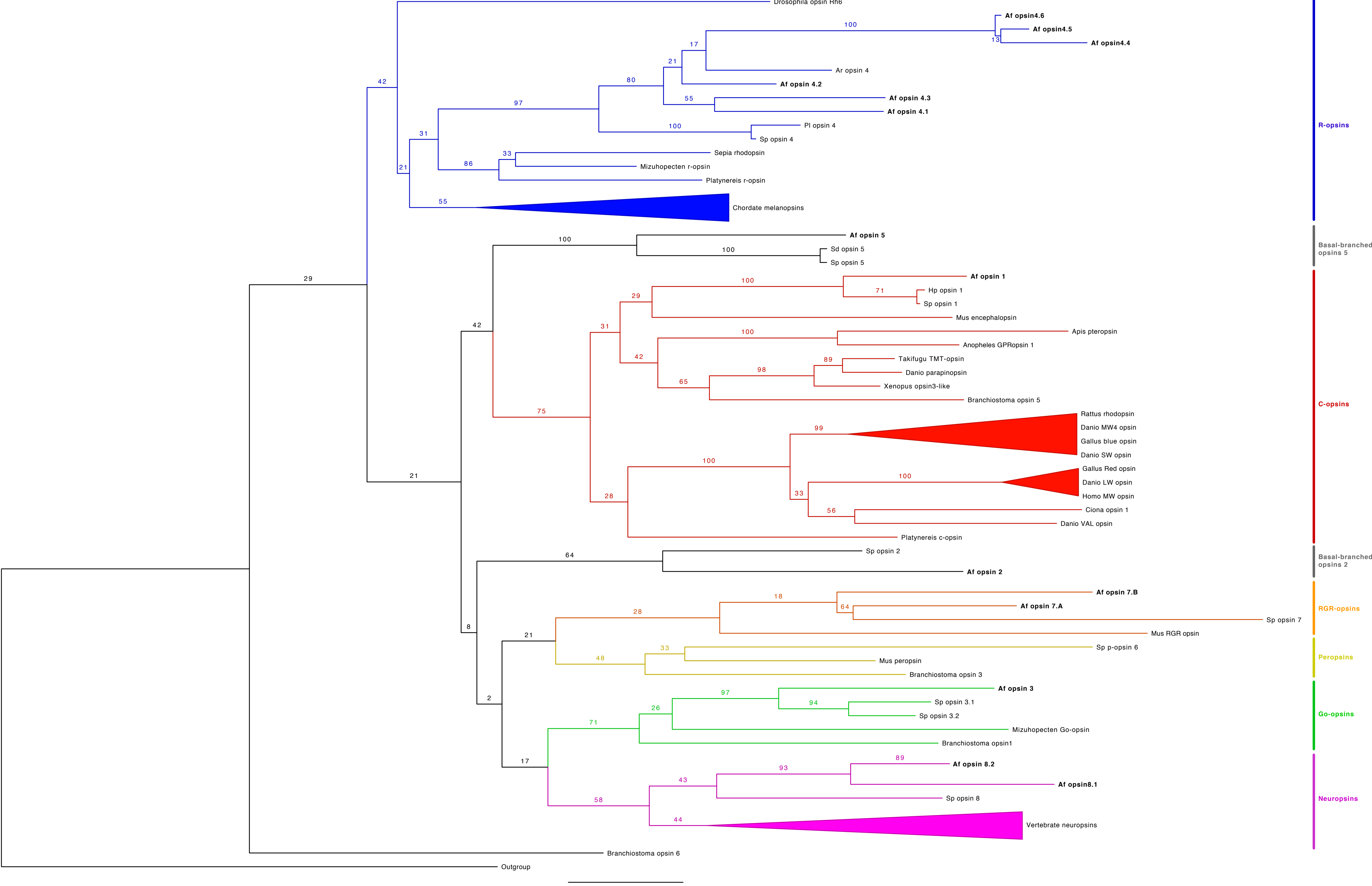

Supplement: Supplementary file 6 — Additional file 6: Phylogenetic tree of metazoan opsins, including the new opsins from Amphiura filiformis, obtained using maximum likelihood inference. Branch length scale bar indicate relative amount of amino acid changes. Branch support values, corresponding to bootstrap proportions, are shown next to the branching points. A. filiformis opsins are represented in bold (Af). Other echinoderm opsins were included in the analyses: Strongylocentrotus purpuratus (Sp), Strongylocentrotus droebachiensis (Sd), Paracentrotus lividus (Pl), Hemicentrotus pulcherrimus (Hp), Asterias rubens (Ar). (PDF 416 KB) [file 12864_2014_6862_MOESM6_ESM.pdf]

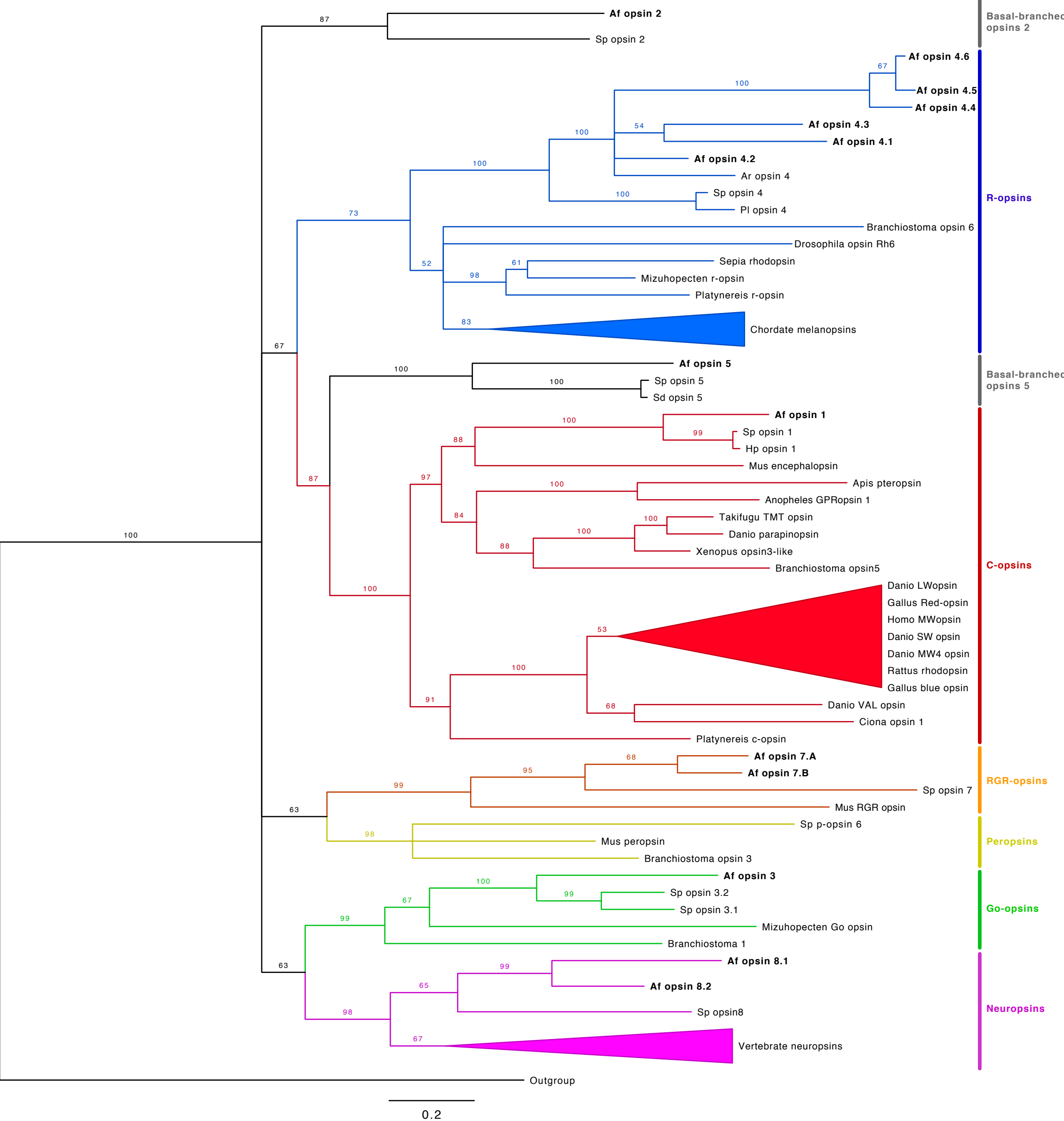

Supplement: Supplementary file 7 — Additional file 7: Phylogenetic tree of metazoan opsins, including the new opsins from Amphiura filiformis, obtained using Bayesian inference. Branch length scale bar indicate relative amount of amino acid changes. Branch support values, corresponding to Bayesian posterior probability, are shown next to the branching points. A. filiformis opsins are represented in bold (Af). Other echinoderm opsins were included in the analyses: Strongylocentrotus purpuratus (Sp), Strongylocentrotus droebachiensis (Sd), Paracentrotus lividus (Pl), Hemicentrotus pulcherrimus (Hp), Asterias rubens (Ar). (PDF 216 KB) [file 12864_2014_6862_MOESM7_ESM.pdf]
